# Milestones in Personalized Medicine in Pemphigus and Pemphigoid

**DOI:** 10.3389/fimmu.2020.591971

**Published:** 2021-01-11

**Authors:** Katja Bieber, Khalaf Kridin, Shirin Emtenani, Katharina Boch, Enno Schmidt, Ralf J. Ludwig

**Affiliations:** ^1^ Lübeck Institute of Experimental Dermatology and Center for Research on Inflammation of the Skin, University of Lübeck, Lübeck, Germany; ^2^ Department of Dermatology and Center for Research on Inflammation of the Skin, University of Lübeck, Lübeck, Germany

**Keywords:** precision medicine, pemphigus, pemphigoid, diagnosis, treatment

## Abstract

Pemphigus and pemphigoid diseases are autoimmune bullous diseases characterized and caused by autoantibodies targeting adhesion molecules in the skin and/or mucous membranes. Personalized medicine is a new medical model that separates patients into different groups and aims to tailor medical decisions, practices, and interventions based on the individual patient`s predicted response or risk factors. An important milestone in personalized medicine in pemphigus and pemphigoid was achieved by verifying the autoimmune pathogenesis underlying these diseases, as well as by identifying and cloning several pemphigus/pemphigoid autoantigens. The latter has become the basis of the current, molecular-based diagnosis that allows the differentiation of about a dozen pemphigus and pemphigoid entities. The importance of autoantigen-identification in pemphigus/pemphigoid is further highlighted by the emergence of autoantigen-specific B cell depleting strategies. To achieve this goal, the chimeric antigen receptor (CAR) T cell technology, which is used for the treatment of certain hematological malignancies, was adopted, by generating chimeric autoantigen receptor (CAAR) T cells. In addition to these more basic science-driven milestones in personalized medicine in pemphigus and pemphigoid, careful clinical observation and epidemiology are again contributing to personalized medicine. The identification of clearly distinct clinical phenotypes in pemphigoid like the non-inflammatory and gliptin-associated bullous pemphigoid embodies a prominent instance of the latter. We here review these exciting developments in basic, translational, clinical, and epidemiological research in pemphigus and pemphigoid. Overall, we hereby aim to attract more researchers and clinicians to this highly interesting and dynamic field of research.

## Pemphigus and Pemphigoid

Pemphigus and pemphigoid are autoimmune diseases of the skin and/or mucous membranes characterized and caused by autoantibodies targeting structural proteins (1). In individual patients, the specific pemphigus or pemphigoid disease is diagnosed based on the clinical presentation, the detection of tissue-bound autoantibodies and the autoantibody specificity (2–4). Albeit rare, pemphigus and pemphigoid diseases impose a major disease burden with a high unmet medical need (5, 6). The clinical hallmark of pemphigus and pemphigoid is (muco)-cutaneous blistering, which occurs intradermal in pemphigus and subepidermal in pemphigoid. In both diseases, autoantibodies are generated in a CD4-dependent fashion. As a general principle, blistering occurs directly as a result of autoantibody binding to the target antigens and *via* complement-independent mechanisms in pemphigus, whereas blistering in pemphigoid usually depends on the activation of innate immune responses through the Fc-portion of the autoantibodies (7, 8). Pemphigus is currently treated with high-dose corticosteroids and the anti-CD20 antibody rituximab, which achieves complete remissions in 80% of the patients (9). The most effective treatment of bullous pemphigoid (BP), by far the most common pemphigoid disease, is long-term application of superpotent topical or oral corticosteroids (10). Epidermolysis bullosa acquisita (EBA) is another pemphigoid disease characterized by a chronic course and is often more refractory to treatment as compared to BP. The main challenges in treating pemphigus are the relative long time needed to induce remissions, high rate of adverse events, and relapse after stopping treatment (11). In BP, relapses after cessation of treatment are the main challenge, as these leads to prolonged treatment with corticosteroids, which are partially responsible for the increased morbidity and mortality of the patients (12).

**Figure 1:**
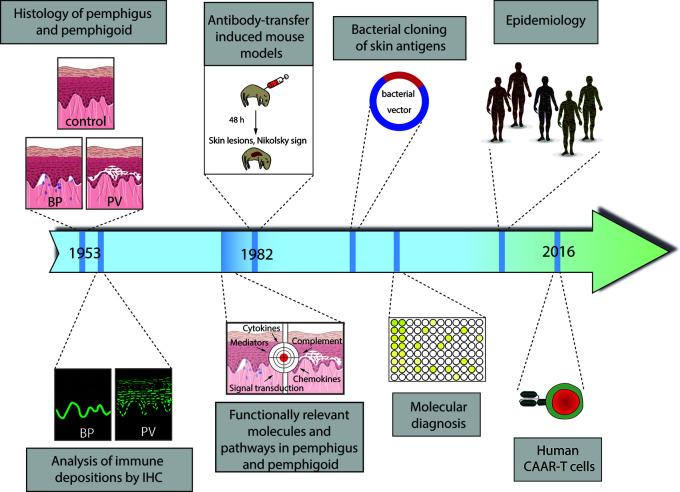
Schematic diagram of the milestones in personalized medicine in pemphigus and pemphigoid. (1) Distinction of pemphigus and pemphigoid based on clinical observation and histology of lesional skin. (2) Identification of distinct binding patterns of autoantibodies in pemphigus (exemplified by pemphigus vulgaris, PV) and pemphigoid (exemplified by bullous pemphigoid, BP). (3) Proof of the autoimmune pathogenesis of pemphigus and pemphigoid by transfer of patient IgG into mice with the subsequent development of pemphigus. (4) Identification and cloning of autoantigens. (5) Establishment of the current, molecular-based diagnostics of pemphigus and pemphigoid. (6) Identification of unique subgroups by careful clinical observation and epidemiology. (6) Individualized treatment by the use of chimeric autoantigen receptor (CAAR) T cells. More details are given in the text.

The current research on pemphigus and pemphigoid diseases is, in our opinion, based on the landmark discovery by Walter Lever in 1953, who, for the first time, clearly differentiated between pemphigus and pemphigoid diseases, mainly based on the histology of skin affected by either one of the diseases (13). This differentiation between pemphigus and pemphigoid based on lesional histopathology promoted tailoring specific treatments for patients with autoimmune bullous diseases (AIBDs), as patients with pemphigus necessitated more aggressive immunospuressive therapy as compared to their counterparts with BP. Subsequently, further milestones in personalized medicine in pemphigus and pemphigoid were made (**Figure 1**):

1. Identification of distinct patterns of autoantibody deposits in the skin
2. Discovery of unique autoantigens in distinct pemphigus and pemphigoid diseases
3. Defining pemphigus and pemphigoid as autoimmune diseases
4. Establishing the current, molecular-based diagnosis of pemphigus and pemphigoid
5. Exploring the functionally relevant molecules and pathways by the use of complex model systems
6. Defining unique pemphigus and pemphigoid variants based on epidemiology
7. Moving towards personalized treatment, selectively targeting specific, autoreactive B cells

These milestones in personalized medicine in pemphigus and pemphigoid are presented in detail in the following sections.

## Histological Differentiation Between Pemphigus and Pemphigoid

**Figure 2:**
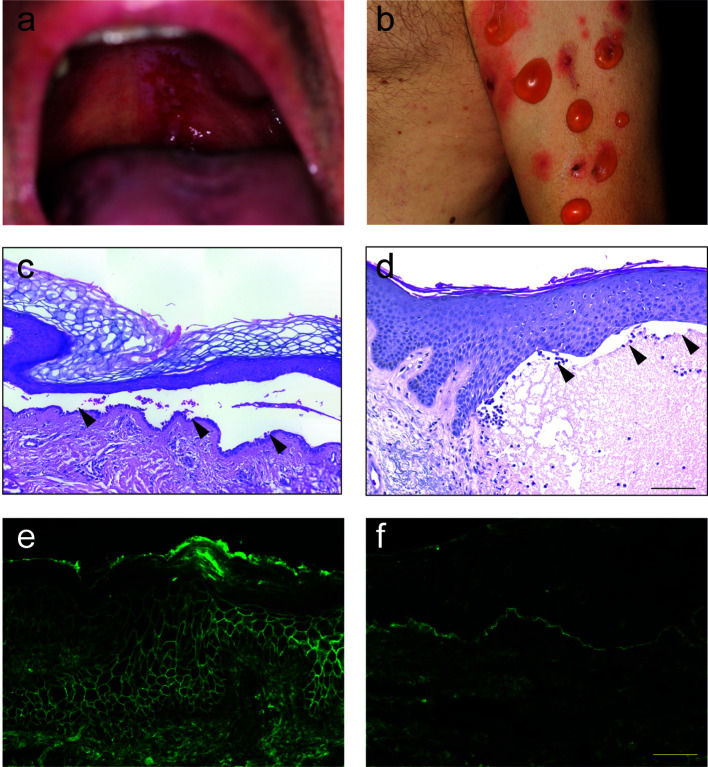
Clinical, histological and immunological features of pemphigus and pemphigoid. **(A)** Clinical presentation of pemphigus vulgaris with erosions at the upper palate. **(B)** Clinical presentation of bullous pemphigoid with blistering at erythematous or otherwise apparently healthy skin. **(C)** Lesional histopathology of a patient with pemphigus vulgaris showing suprabasal splitting with some acantholysis and the typical “row of tombstones” (arrows). **(D)** In a lesional biopsy of a patient with bullous pemphigoid, subepidermal splitting. In this case, interestingly, the dermal infiltrate is only marginally present. **(E)** Tissue-bound IgG in a perilesional skin biopsy of a pemphigus vulgaris patient, showing IgG deposits in a honeycomb-like pattern within the epidermis. **(F)** Tissue-bound IgG in a perilesional skin biopsy of a bullous pemphigoid patient, showing linear IgG deposits along the dermal-epidermal junction.

The term “pemphigus” was used as descriptive terms for skin diseases characterized by blisters since Hippocrates (460-370 B.C.) who described different types of fever associated with blistering as “pemphigus fever”. However, the term pemphigus in its present meaning was coined by Dr. Wichman in 1791 when describing a case of pemphigus (in today’s understanding). Thereafter, “pemphigus” was used as a synonym for any vesicular or bullous disease. This led to the emerge of several different types of “pemphigus” (14). In 1860, Dr. Hebra reinstated the concept established by Wichman, stating that “pemphigus” is always a chronic disease. Thus, all pemphigus and pemphigoid diseases were subsumed under the term “pemphigus” (15). Based on the prognosis, two different forms of “pemphigus” were differentiated: Malignant and benign pemphigus. In 1953, Walter Lever published his landmark histological observations where he distinguished between pemphigus, characterized by intraepidermal blistering, and BP, characterized by subepidermal blistering (13) (**Figure 2**). Taking the clinical presentation into account, he also coined the term mucous membrane pemphigoid (MMP) that is characterized with a histology similar to BP but with blistering at mucosal sites (13). Thus, Walter Levers’ careful clinical and histological observations still hold true and are a good example of how thorough clinical observations allow landmark discoveries.

## Identification of Unique Autoantibody Deposits in Pemphigus and Pemphigoid

About two decades after the discovery of Dr. Lever, IgG deposits were noted in patients with pemphigus (16) and pemphigoid (17). In pemphigus, immunoglobulin (Ig) deposits were found to be located at the surface of the keratinocytes. Thus, the term honeycomb-like fluorescence observed within the epidermis has been coined for this particular staining pattern (**Figure 2**). In pemphigoid diseases, linear Ig/complement (C) deposits along the dermal-epidermal junction are observed. For this, staining pattern the term “linear” Ig/C deposition has been established (**Figure 2**). More recently, unique patterns of IgG deposits have been observed in pemphigoid diseases. In contrast to all other pemphigoid diseases where the IgG deposits show a “n-serrated” pattern, the pemphigoid disease EBA displays an “u-serrated” pattern of both IgG and IgA (18–20). The identification of these patterns in direct immunofluorescence (IF) microscopy is a learnable skill (21), which is essential for the diagnosis of EBA because up to 60% of cases are seronegative (22). The observation of these unique staining patterns in pemphigus and pemphigoid (as well as in EBA) supported the histopathological observation in 1953 that pemphigus and pemphigoid are distinct diseases.

## Defining Pemphigus and Pemphigoid as Autoimmune Diseases

The deposition of IgG in the skin suggested that pemphigus and pemphigoid may be caused by an immune response to self-antigens. Yet, direct proof of the autoimmune pathogenesis of pemphigus and pemphigoid were missing. According to the revised Witebsky’s postulates, such direct proof is the induction of the disease in experimental animals by transfer of patient serum or IgG (23). The autoimmune nature of pemphigus was demonstrated by Grant Anhalt and colleagues in 1982, when they induced skin blistering and erosions, accompanied by the histologic, ultrastructural, and immunological findings found in pemphigus patients, by transfer of pemphigus patient IgG into neonatal Balb/c mice (24). Attempts to reproduce the pemphigoid disease BP by transfer of patient IgG into mice failed because the transferred IgG did not bind to the skin of the mice (25). Several years later, in 1993, when the autoantigens in BP had been defined (BP180 and BP230), and the lack of cross-reactivity of human autoantibodies with murine BP180 had been demonstrated, Zhiou Liu and colleagues were able to induce experimental BP in mice by the transfer of rabbit anti-mouse BP180 IgG (26). Collectively, the defining of pemphigus and (later) pemphigoid as autoimmune diseases, and the demonstration of distinct clinical, histological and immunological features in experimental pemphigus and pemphigoid triggered the search for the autoantigens. Subsequently, the pathogenic relevance of several autoantigens and their corresponding antibodies has been demonstrated in different mouse models for pemphigus foliaceus (PF), paraneopalastic pemphigus (PNP), MMP, and EBA.

## Identification of Distinct Autoantigens in Pemphigus and Pemphigoid

Identification of Ig deposits in the skin, as well as the proof of the autoimmune pathogenesis of pemphigus and pemphigoid initiated the search for putative autoantigens, which is still ongoing; i.e., in the case of anti-p200 pemphigoid (27). Using patient autoantibodies, and monoclonal antibodies against type VII collagen (COL7), COL7 was identified as the autoantigen in EBA, as early as in 1988 (28). In 1990, BP180 was cloned human from a keratinocyte library (29). One year later, both, desmoglein (Dsg) 1 and 3 were cloned and identified as the autoantigens in PF and pemphigus vulgaris (PV) (30–32). Subsequently, additional autoantigens in pemphigus and pemphigoid were cloned and/or identified (**Table 1**).

**Table 1:** Autoantigens in pemphigus and pemphigoid.

	Disease	Main target antigen(s)	Main isotype(s)
**Pemphigus**	**Pemphigus vulgaris (PV)**	Dsg3 in mucosal PV, Dsg 1 and 3 in muco-cutaneous PV	IgG
	**Pemphigus foliaceus (PF)**	Dsg 1	IgG
	**Paraneoplastic pemphigus (PNP)**	Envoplakin, periplacin, Dsg 1/3, BP180, and others	IgG
	**IgA pemphigus**	Dsg 1/3, Dsc 1-3	IgA
	**Endemic pemphigus foliaceus**	Dsg 1	IgG
	**Herpetiform pemphigus**	Dsg 1	IgG
**Pemphigoid**	**Bullous pemphigoid (BP)**	BP180-NC16A, BP230	IgG/IgE
	**Mucous membrane pemphigoid (MMP)**	BP180, BP230, laminin-332, α4β6 integrin, laminin-331, COL7	IgG
	**Pemphigoid gestationis (PG)**	BP180-NC16A	IgG
	**Linear IgA disease (LAD)**	LAD-1	IgA
	**Epidermolysis bullosa acquisita (EBA)**	COL7	IgG/IgA
	**Anti-.p200 pemphigoid**	Laminin γ1 (non-pathogenic autoreactivity)	IgG
	**Lichen planus pemphigoides**	BP180-NC16A, BP230	IgG

Please note that pemphigus may also be induced by non-Dsg autoantibodies (33, 34). However, in >95% of pemphigus vulgaris/foliaceus patients, anti-Dsg autoantibodies are present (35, 36). Thus, non-Dsg autoantibodies were not included in this table. Dsg, desmoglein; Dsc, desmocollin; COL7, type VII collagen; LAD-1, linear IgA disease antigen-1 (soluble ectodomain of BP180).

## Molecular-Based Modern Diagnosis of Pemphigus and Pemphigoid

Definition of distinct autoantigens in pemphigus and pemphigoid diseases enabled the development of the currently used, molecular-based diagnosis of pemphigus and pemphigoid (37). If clinically suspected, the detection of tissue-bound autoantibodies (or C3) in a perilesional skin (or mucosal) biopsy is the gold standard for the diagnosis of pemphigus and pemphigoid. Depending on the location of the Ig or C3 deposits, pemphigus (deposits in the intercellular space, also termed honeycomb pattern), or pemphigoid (linear staining along the dermal-epidermal junction) are differentiated. EBA can be further differentiated based on the serration pattern (19). This pattern analysis is a learnable and important skill because EBA may be seronegative in 60% of the cases (21, 22).

If the diagnosis cannot be established based on the direct IF microscopy, indirect IF microscopy using different organ substrates, most frequently monkey esophagus and human salt-split skin, can further differentiate between the different pemphigus and pemphigoid diseases (37). Other less frequently utilized substrates are rat bladder for PNP and normal oral mucosa for MMP (38). While monkey esophagus is useful in the detection of circulating pemphigus-related autoantibodies, pemphigoid autoantibodies better bind to salt split skin. Of note, if a linear deposit of patient autoantibodies is observed at the roof of the blister of the artificially split skin, BP (or MMP in patients with predominant mucosal involvement) is diagnosed because its autoantigens (BP180 and BP230) are expressed at the blister roof. By contrast, laminin-332, COL7 and p200 are expressed at the blister floor. Hence, binding of patient autoantibodies to the blister floor in indirect IF microscopy on salt split skin requires further differentiation between MMP, EBA, and anti-p200 pemphigoid. The latter, as well as (semi)-quantitative determination of circulating autoantibody concentrations, can be achieved by detection of specific autoantibodies. This can be achieved using the recombinant immunodominant domains of the target antigens, i.e., Dsg1, Dsg3, envoplakin, BP180, BP230, laminin 332, and COL7 in commercial ELISA systems or biochip mosaics. In addition, specialized laboratories have established techniques (mainly Western blotting or immunoprecipitation) for the detection of autoantibodies against p200, selected chains of laminin-332, the ectodomain of BP180, or rare autoantigens. Thus, this molecular-based modern diagnosis of pemphigus and pemphigoid allows (in most cases) to diagnose individual pemphigus and pemphigoid diseases. As their treatment and prognosis greatly differs (2, 3), this allows to select the appropriate treatment for each patient. In addition to their use in diagnosis, longitudinal monitoring allows for early detection of relapses because circulating autoantibody concentrations correlate intraindividually with disease activity (39–41). Molecular characterization of some AIBD, however, is still possible only in highly specialized academic centers. A possible consequence is the delay experienced by patients in receiving the right diagnosis and optimal treatment.

A more recent development in the personalized management of pemphigoid is the identification of biomarkers other than the autoantibodies that allow to predict treatment response and/or relapse (42). In brief, persistence of elevated eosinophil cationic protein (ECP) in patients with BP was associated with relapse (43). In addition to persistence of elevated ECP levels, the presence of anti-COL7 autoantibodies (the autoantibody in EBA) (44), and increased CXCL10 serum levels (45) are also predictors of relapse in patients with BP. Relative to non-autoreactive B cells, autoreactive B cells of patients with PV showed overexpression of genes encoding for IL-1β, IL-23p19, and IL-12p35 pro-inflammatory cytokines and the IRF5 transcription factor. Relative to patients with active pemphigus, those experiencing complete remission following rituximab displayed under-expression of IL-1β and the CD27 memory marker genes (46).

Efforts were extensively made to establish a personalized approach to optimize management of patients with pemphigus. That is, to predict patients predisposed to early relapses under rituximab who may benefit from maintenance rituximab infusion at month 6. Increased severity score at baseline and increased levels of anti-Dsg1 and anti-Dsg3 three months following the first infusion were found to independently project post-rituximab early relapse, thus warranting to consider a maintenance dose of rituximab after 6 months (47).

## Functionally Relevant Molecules and Pathways in Pemphigus and Pemphigoid

Employing keratinocyte cultures, *ex vivo* skin models, and the above-mentioned mouse models (48, 49), several disease pathways have been identified that provide the base for valuable novel therapeutic approaches. Dsgs are transmembrane desmosomal cadherin-like glycoproteins which function to maintain tissue integrity and facilitate cell-cell adhesion. IgG autoantibodies targeting Dsg3 and Dsg1 play the main etiopathogenetic role in the development of PV and PF, respectively. In pemphigus, monovalent fragments of anti-Dsg antibodies that lack the Fc portion are sufficient to cause acantholysis *in vitro* and *in vivo* (50). The exact sequence of events in anti-Dsg antibody-mediated acantholysis has not yet been fully understood. Three major events following the binding of anti-Dsg IgG have been described: (i) direct interference with Dsg transinteraction, a phenomenon termed steric hindrance, (ii) remodeling of Dsg expression on the cell surface leading to internalization and depletion of Dsg from the cell membrane, and (iii) signaling events that impair cytoskeletal architecture (3, 4). These mechanisms do not apply equally for Dsg1 IgG- and Dsg3 IgG-binding. Upon targeting of Dsg1, Ca^2+^ influx is induced and the ERK pathway is activated. In contrast, after binding of Dsg3-specific IgG, signaling *via* p38MAPK occurs in the epidermis but not in mucosal tissues, and SRC family of protein tyrosine kinases and EGFR pathways are activated (51, 52). Current data also strongly suggest that, in addition to Dsg1/3 autoantibodies, non-Dsg antibodies, as well as soluble Fas ligand contribute to the pemphigus phenotype (53–56).

In contrast to pemphigus disorders, in pemphigoid diseases, FcγR-mediated effects are pivotal for blister formation, and over the last decade several disease pathways and key molecules with functional relevance in these diseases have been described including several signaling molecules, leukotriene B4 (LTB4), and IL-17 (57–60). Furthermore, complement activation at the dermal-epidermal junction is generally accepted to be a cornerstone in recruiting neutrophils, eosinophils, and macrophages to this site (61). Of note, subtle differences in the impact of complement activation emerged between different pemphigoid diseases as well as between clinical variants of BP. In contrast, acantholysis in pemphigus appears to develop independently of complement activation although staining of C3 in the epithelium/epidermis is a diagnostic hall mark (3). Exploring these differences may uncover patient and diseases subgroups that can benefit from therapeutic interventions in targeting complement components. About 80% of BP patients reveal C3c deposition along the dermal-epidermal junction in perilesional biopsies. In patients with C3c deposition, anti-BP180 NC16A IgG serum levels were significantly higher and patients without blisters had significantly less C3c deposits along the dermal-epidermal junction (62). While no relation between the extent of skin lesions and C3c staining in the skin of patients was found, the complement activation capacity of autoantibodies in the *ex vivo* complement fixation assay correlated with diseases activity as measured by the Bullous Pemphigoid Disease Area Index (BPDAI) (63). Further support for the relevance of complement activation in BP comes from the finding of elevated levels of C3a in the serum of BP patients and the positive correlation of serum C3a levels with both anti-BP180 NC16A IgG and soluble CD46, a crucial complement regulatory protein in the complement activation (64). However, plasma concentrations of C3a, C4a, as well as C5a are not different between BP patients and age/sex matched controls. Furthermore, the plasma levels of these three complement components remain constant when evaluated in flares and in remission of BP (65). In the neonatal BP mouse model, where blisters typically develop 24–48 h after injection of rabbit IgG against the NC15A domain of BP180, the blistering phenotype was completely dependent on complement activation at the dermal-epidermal junction (66, 67). The same complement dependency was observed in a humanized mouse model of BP in which the human NC16A domain replaced the homologous murine NC15A region (68). In contrast, in another humanized mouse model of BP, in which the entire BP180 molecule had been replaced by the human protein, injection of polyclonal F(ab’)2 anti-NC16A IgG or non-complement-activating anti-NC16A IgG4 led to blister formation (69, 70). In line, Dainichi et al. reported on two BP patients without C3 deposits in the skin and IgG4 autoantibodies as main subclass that were unable to elicit complement activation *ex vivo* (71). Additional observations in this model with C3-deficient animals that were susceptible to the pathogenic effect of anti-BP180 IgG and recently, in a BP model in adult mice where transfer of anti-BP180 NC15A IgG in C5aR1-deficient mice led to a reduction of skin lesions by 50% (70, 72, 73) pointed to complement-independent mechanisms in BP pathophysiology. Interestingly, similar to the BP neonatal mouse model, in adult mouse models of EBA and anti-laminin 332 MMP, complement activation appeared as prerequisite for a blistering phenotype (74–76). Unravelling the complex scenario of complement activation in pemphigoid disorders will certainly help to identify patient subpopulations and to tailor more specific and safe treatments for these diseases. The dose-dependent inhibition of the BP IgG-induced C3 deposition at the dermal-epidermal junction in the *in vitro* complement fixation on cryosections of human skin by (i) the anti-C1s antibody TNT003, (ii) the low-molecular-weight heparin tinzaparin sodium, and (iii) the dual C5/LTB4 antagonist coversin, all of the which disturbing the normal activity of complement pathway (65, 77, 78) directs us to further explore complement inhibition as valuable therapeutic target in BP.

## Epidemiology Defines Unique Variations Within Several Pemphigus and Pemphigoid Diseases

As its name indicates, PV is the most prevalent subtype of pemphigus, comprising up to 70% of all cases (79). PV is typified by a variable geographic and ethnic distribution, with annual incidence rates ranging between 0.8 and 16.1 cases per million population in different regions (80). Congruently, a predisposition for developing pemphigus was reported in some ethnic groups, namely, Ashkenazi Jews and individuals of Mediterranean origin (80). In a recent population-based study, the incidence of PV was 3.6-fold increased among Jews as compared to Arabs in Israel (81). In a retrospective study conducted in Connecticut, the US, the annual incidence of PV was almost eight-fold higher among people of Jewish ancestry than among those belonging to other ethnic groups (82). These epidemiological observations have been strongly substantiated by genetic studies disclosing an association of several HLA-class II genes, HLA-DRB1*04, and HLA-A*10, with the occurrence of PV among Ashkenazi Jews (83–85). Subsequently, a polymorphic variant in *ST18* gene was associated with PV in Jewish and Egyptian but not in German patients (86). Hence, despite an underlying genetic pre-disposition in PV, the disease may manifest also in the absence of certain genetic predisposing factors. Collectively, this points towards a polygenic genetic risk to develop PV, as well as points towards the environment as a potential driver of disease pathogenesis, as reported for other inflammatory diseases (87).

Sporadic PF is an uncommon disease constituting 20%–30% of all pemphigus cases. Its estimated annual incidence in Europe and the United States is less than one case per million population (80). HLA-DRB1*04 was associated with increased risk for sporadic PF in Brazilian, Italian, French, and Dutch populations (88, 89). An association with HLA-DRB1*0101 was found among patients with sporadic PF originating from Mexico (90). Nevertheless, no ethnic predilection in the occurrence of PF was noted in Israel, as the adjusted incidence rate of the disease was comparable between Jews and Arabs (81). Apart from sporadic cases, endemic subtypes of PF have been described in Brazil (*folgo selvagem)*, Colombia, and Tunisia (80). Although patients with endemic PF are clinically, histologically, and immunologically indistinguishable from those with sporadic PF (80, 91), the former is characterized by a patterned geographic distribution, familial predisposition, and younger age of presentation (92, 93).

BP is the most common subepidermal AIBDs worldwide. BP characteristically affects the elderly and is seen mainly among patients older than 75 years. While the general annual incidence of BP has been reported to range between 2.4 and 23.0 cases per million population, it rises exponentially to 312 cases per million population in individuals older than 80 years (94). Several lines of evidence accumulated to suggest a notable increasing incidence of BP by 1.9 to 4.3 folds over the past two decades (95). Several putative interpretations were postulated to account for this observation, the most plausible of which is the growing exposure to certain medications implicated with the induction of BP (95). Dipeptidyl-peptidase IV inhibitors (DPP4i), also known as gliptins, a recently introduced anti-diabetic class of oral medications, emerged as a potential trigger of BP (96–98). It is yet to be decisively determined whether patients with DPP4i-associated BP follow a distinct clinical and immunological profile. While European studies did not depict distinct features for these patients distinguishing them from typical BP, DPP4i-associated BP patients originating from Japan were more likely to present with non-inflammatory BP and to target the immunodominant domain of BP180 antigen (NC16A) less frequently (99–101). Thus, careful clinical observation has led to the identification of a unique clinical presentation of BP. Given that the initial observations of unique immunological and genetic features of DPP4i-asscociated BP can be confirmed, this would allow to treat the patients according to the underlying, disease-promoting pathways. In the case of DPP4i-associated BP, this is relatively easy because BP usually clears after stopping DPP4i treatment.

## CAAR T Cells as a Potential and Personalized Cure of Pemphigus and Pemphigoid

The most recent advance towards a personalized treatment approach is the development of chimeric autoantigen receptor (CAAR) T cells for the treatment of pemphigus (102). Based on the breakthrough discovery of chimeric antigen receptor (CAR) T cells for the treatment of hematologic malignancies (103), Aimee Payne and colleagues developed a recombinant T cell receptor by fusing the autoantigen in PV (Dsg3) to CD137-CD3ζ. These Dsg3 CAAR T cells exhibited specific cytotoxicity against B/plasma cells expressing the B cell receptor specific for Dsg3. Next, experimental pemphigus was induced in mice by transfer of Dsg3-hybridoma cell lines. When mice were additionally injected with Dsg3 CAAR T cells, they were protected from induction of experimental pemphigus (102). Subsequent work from the same group expanded the work to additional autoantigens in pemphigus, namely, Dsg1 (104). Currently, a phase I clinical trial is conducted on autologous Dsg3 CAAR T cells in mucosal PV (https://cabalettabio.com/clinical-trials/, accessed May 31, 2020). By selectively targeting autoreactive B cells, using the CARR T cell technology, a highly personalized treatment approach for PV is currently under development (105). Overall, given successful completion of this (and subsequent) clinical trials, a new era of managing B cell-driven autoimmune diseases (106) will emerge.

## Future Perspectives

The emergence of treatments selectively targeting autoreactive B cells, e.g., by CAAR T cells is expected to significantly change the treatment of pemphigus. It is highly intriguing to estimate whether CAAR T cells the same potential additionally in pemphigoid diseases. Alternatively, in another approach, immunization of PV patients with Dsg3-coated nanoparticles may specifically suppress autoimmunity against Dsg3 and is currently performed in a phase I clinical trial in PV with Dsg3. Targeting IL-17 and eotaxin is currently being assessed as a potential therapeutic approach in BP (107). The success of these treatments will, however, depend on an expansion of the molecular diagnosis that will allow to precisely define the autoimmune response in individual patients. In parallel, molecular diagnosis is also expected to help define distinct (sub)-groups of pemphigus and pemphigoid diseases that most likely will be more and more based on molecular signatures. In the long term, one may envision that curative and safe treatments for pemphigus and pemphigoid will be available.

## Author Contributions

KBi, KK, SE, KBo, ES, and RL wrote the manuscript. All authors contributed to the article and approved the submitted version.

## Funding

Excellence Cluster “Precision Medicine in Chronic Inflammation” (EXC 2167) from the Deutsche Forschungsgemeinschaft.

## Conflict of Interest

During the last 3 years, ES has received research funding from Novartis, UCB, Incyte, Biotest, Euroimmun, Dompe, Admirx, Synthon, TxCell, ArgenX, and Fresenius Medical Care and fees for consulting or speaking and/or travel expenses from Novartis, UCB, Biotest, TxCell, ArgenX, Roche, Genentech, Imevax, Amryth, Thermo Fisher, AstraZeneca, True North Therapeutics, Bristol-Myers Squibb, and Fresenius Medical Care. RL has received research funding from Almirall, True North Therapeutics, UCB Pharma, ArgenX, TxCell, Topadur, Incyte, and Admirx and fees for consulting or speaking from ArgenX, Immunogenetics, Novartis, and Lilly.

The remaining authors declare that the research was conducted in the absence of any commercial or financial relationships that could be construed as a potential conflict of interest.
